# An Essay on the Application of Lunar Caustic in Certain Wounds and Ulcers

**Published:** 1826-04

**Authors:** 


*An Essay on the Application of the Lunar Caustic, in the Cure of certain Wounds and Ulcers.* By JOHN HIGGINBOTTOM, Nottingham, Member of the Royal College of Surgeons of London. 8vo, pp. 147. London, January, 1826.

THIS little opuscule is dedicated to the author's brother-in-law, Dr. Marshall Hall, and appears to have been constructed in the same school of minute clinical observation, in which the latter has so ably and usefully laboured. As a direct appeal to facts, there is but little scope for criticism, in reviewing such a production. If writers, indeed, adhered to the plan adopted by Mr. Higginbottom, critics would soon have little to do, and the war of words which rages in the literary, would soon be as still and hushed as the war of cannon in the military world. How far such an armistice might be beneficial to the interests of science, is a question not so easily solved; but certainly such an event would save a tremendous effusion of ink —reduce the rent of *attics*—and send their tenants, the genii of invention, to the work-house, or, perhaps, to the tread-mill.

Mr. Higginbottom presents his observations to the public, with very humble pretensions. "It is chiefly with the minor accidents or diseases that they have to do"—but let it be remembered that—" nihil est aliud magnum quam multa minuta." We shall consider that he has not laboured in vain, if he is enabled to mitigate even these little evils of human life.

Lunar caustic has been long a very favourite application, in the hands of our best surgeons, to ulcers of an irritable, as well as of an indolent nature. Indeed, we have seen the most admirable effects from its use, not only in affections of the cutaneous surface, but in irritable states and conditions of the throat, and even of the larynx. A sponge dipped in a weak solution of the argentum nitratum, and secured as in a probang, may be applied to the fauces with great advantage, in irritation of the epiglottis, larynx, and neighbouring parts, as well as in ulcerations of the same. This remedy is not so well known nor so often put in practice as it deserves to be. Mr. H. has certainly extended the use of the lunar caustic, in external affections, beyond any other surgeon that we know of, and we shall, therefore, exhibit a more detailed analysis of this little volume than its size would otherwise warrant, convinced that we shall thereby enlarge the sphere of its utility.

The work consists of three chapters—namely, on healing by eschar—on the application of this mode of treatment to different cases—and, on some cases in which the caustic is inapplicable.

Chap. 1. *Healing by Eschar.* Every one is familiar with the effects of lunar caustic applied to an abraded surface—first, a white film, assuming, in a few hours, a darker colour—then a degree of hardening, resembling a bit of sticking-plaster—and, finally, in a few days, a corrugation and gradual separation of the film, or eschar, leaving the subjacent surface of the sore healed. To form this eschar properly, the caustic should be applied to the whole surface of the sore, and even a little beyond its limits, otherwise the contraction of the eschar may leave a space between its edges and the healthy skin. In recent wounds, unattended by inflammation, the caustic may be applied freely—but when inflammation has come on, too severe an application of the caustic induces vesication of the surrounding skin, and loosening or removal of the eschar. If every part is touched, a slight application of the caustic is generally sufficient. Mr. H. avers, from repeated and extensive trials, that —"in every instance in which the eschar remains adherent from the first application, the wound or ulcer over which it is formed, invariably heals." On this account, the importance of avoiding all causes which may detach the edges of the eschar, becomes obvious. When the eschar does begin to separate from the healed edges of the sore, it should be carefully removed by a pair of scissars. To preserve the eschar adherent for a proper time, it is only necessary to cover it with a piece of gold-beater's skin, moistening the adjacent surface with a little water to make it adhere. It is easily removed at any time, by again moistening it with water, and may be re-applied in the same manner.

Some other effects of the caustic are now to be mentioned. In the first place, irritability and pain, as in superficial wounds along the skin, are removed by this application. A blush of inflammation forms round the eschar, but this soon subsides, and the inflammation that would otherwise have been set up, is prevented. If inflammation has been established previously, it is increased, at first, by the caustic—but if not severe, and if the eschar remain adherent, all inflammation subsides entirely. When the previous inflammation round the wound is considerable, the caustic would induce vesication, and should not, therefore, be applied. Another mode of treatment, hereafter to be described, is then to be adopted.

The surgical reader will immediately perceive, that this mode of healing, by adherent eschar, is considerably analogous to the well-known natural process of healing under cover of a scab, formed by the dried secretion from the sore, and which has been treated of by Mr. John Hunter. The comparative advantages of the two processes are decidedly, Mr. H. thinks, in favour of the caustic.

"By comparative trials, I have found that whilst the scab is irritable and painful, and surrounded by a ring of inflammation, the adherent eschar is totally free from pain and inflammation; and that whilst the scab remains attended by inflammation and unhealed, the eschar is gradually separating, leaving the surface underneath completely healed. To these observations I may add that the success of the plan of healing by eschar is infinitely more certain as well as more speedy than that by scabbing." 12.

In conclusion, it may be remarked, that the caustic treatment has the recommendation of economy as well as comfort, and is, therefore, of considerable importance in dispensary practice, as well as in that of the army and navy.

*Unadherent Eschar.* The adherent eschar takes place in cases of recent injuries, and in small ulcers, when they are nearly even with the skin and attended by little inflammation. In other cases the eschar is too apt to be unadherent, from the formation of pus or a scab underneath. When pus forms, the centre of the eschar is observed to rise, and to yield to the pressure of a probe. The fluid escapes sometimes by an opening at the side. When a scab forms underneath the eschar, (which does not happen except the fluid has been allowed to remain too long under the eschar without being evacuated) there are pain and some inflammation—the eschar does not separate, but remains long over the sore, and there is no appearance of healing.—When fluid is ascertained to exist under the eschar, a slight puncture is to be made in it—the matter gently pressed out—and the caustic applied over the orifice. The same plan is to be adopted if the fluid ooze out at the edge of the eschar. After this treatment the eschar occasionally remains adherent; but more frequently the fluid requires to be repeatedly evacuated, which should be done every 12 or 24 hours, according to the quantity secreted. If, by accident, the eschar be separated before the sore is healed, Mr. H. recommends the re-application of the caustic.

Where the eschar does not separate favourably, the formation of a *scab* is to be suspected, in which case the whole must be removed by a cold poultice for two or three days, which has the effect, not only of removing the eschar, but of allaying inflammation or irritation. Afterwards the caustic is to be re-applied as before. The gold-beater's skin is still more useful in this than in the case of the adherent eschar; and is to be applied and removed in the manner already described.

The application of the caustic produces a greater or less degree of pain according to the sensibility and size of the wound. It is more severe in recent wounds than in ulcers—but it soon subsides in every case, and then the patient experiences more ease than under any other mode of treatment.

On the other hand, the application of caustic is improper when the ulcer is too large to admit the formation of a complete eschar—where it is so situated as not to remain undisturbed—or where there is much inflammation or œdema. The solid lunar caustic our author has found to be the best—and that which has been made some time is preferable, as not so likely to dissolve and run about.

*Treatment of the Eschar by Poultice.* In many cases where neither the adherent nor the unadherent eschar can be formed, Mr. H. thinks it very useful to apply the caustic first, and then a cold poultice, without lard or oil. This plan is said to be particularly useful in cases of punctured wounds attended by much pain and swelling, and in cases of recently opened abscesses. "By this application the pain and swelling are much subdued, and a free issue is secured for the secreted fluid." In no instance has he seen the original inflammation increased by the caustic. It is necessary to repeat the application every second or third day, the poultice being renewed every eight hours. When the inflammation has subsided, an attempt may be made to form the adherent eschar.

CHAP. II. *Application to particular Cases.* In cases of recent punctured wounds, the orifice and surrounding skin should be moistened with a little water, and the caustic should then be applied within the puncture, until a little pain is felt, and then over the surrounding skin. The eschar is to be allowed to dry. "In this manner it is astonishing how completely the terrible effects of a punctured wound are prevented." The eschar usually remains adherent, and the case requires no further attention. At a later period, when the orifice is nearly closed by inflammation, and when some pus is formed within, the fluid should be pressed out, and the caustic may be applied as before. When it is thought that an abscess of any extent has formed under the puncture, it should be opened freely with a lancet, and treated with caustic and poultice, keeping the latter moist and cold with water.

"In cases of puncture where the orifice is healed and where an erysipelatous inflammation is spreading, attended with swelling, I have applied the caustic freely over and beyond the inflamed parts, and I have had the satisfaction to find that the inflammation has been arrested in its progress and has shortly subsided.

"This mode of treatment is particularly useful in cases of punctured and lacerated wounds from various instruments, such as needles, nails, hooks, bayonets, saws, &c. and in the bites of animals, leech-bites, stings of insects, &c. In considerable lacerations the same objection would exist to this treatment as in large ulcers." 27.

Mr. H. avers, that the dreadful effects of punctures from needles, scratches from bone, or other injuries received in dissection, are totally prevented by this treatment. This averment is corroborated by numerous cases circumstantially detailed by the author, but for the particulars of which we must refer to the work itself.

In respect to the wounds received in dissection, it is not in our author's power to give any cases illustrative of the treatment of the severer accidents of this kind; for since he began the free use of the lunar caustic, all the terrible effects of such wounds have been invariably prevented. In the years 1813 and 1819, Mr. H. was himself exposed to great danger from inoculation, during the examination of dead bodies—and since the latter period, he has been repeatedly exposed to the same danger; but in every instance, the danger has been completely averted by the prompt and free application of the lunar caustic. In recent dissection punctures he would advise the caustic to be applied in the same manner as already described in simple cases of punctured wounds; but when the case has been neglected, and when, as is often the case, a small tumour has formed underneath the skin, attended with smart stinging pain, he advises the tumour to be removed with a lancet, and the caustic to be applied, both to the surface of the wound and to the surrounding skin, so as to form an adherent eschar. In cases still more neglected and advanced, where inflammation of the absorbents has supervened, "a free crucial incision is to be made, the caustic is to be freely applied, and afterwards the cold poultice and lotion, the usual constitutional remedies being actively enforced."

We would here take the liberty of suggesting the propriety of distinguishing those dissection wounds which arise from the introduction of putrid matter into a wound, producing local irritation, inflammation of the absorbents, and certain constitutional symptoms of no very dangerous character, from that formidable train of phenomena which results from a dissection wound, where the body is quite fresh, and where death has resulted from some form of inflammation, as puerperal peritonitis. In this last case, the constitution is often affected in a few hours after the puncture, and while there is scarcely a cognizable mark of the wound. The breathing becomes oppressed—the nervous system disturbed—in short, there is the most presumptive evidence of a morbid poison having been introduced, against which we have systematic practice or plan of treatment. In the first class of dissection wounds, (from putrid matter) we have inflammation of the absorbents and its consequences to obviate, and the treatment is neither obscure nor difficult—but in the second class, (a morbid poison from a recently dead subject) we have a most dangerous disease, the true nature of which we are ignorant of, and the treatment of which is necessarily uncertain, difficult, and too often ineffectual. It is by confounding these two kinds of dissection-wounds that we have such eternal wrangling debates in our medical societies, and literary discrepancies in our journals. It is very clear that in the dangerous class alluded to, we cannot expect to stem the action of the poison by depletion—no more than we could quell the action of variolous matter introduced into the system. All we can do, is to combat the symptoms as they rise. If the nervous system bear the onus, it would be madness to bleed the patient; whereas, if distinct inflammations of internal organs or structures be set up, we must deplete, be the consequences what they may. The effects of the morbid poison are modified by the idiosyncrasy of each individual, and, consequently, the treatment which would be rational and proper in one person, would be death in another, even if the two individuals received the poison at the same moment from the same subject. Hence it follows—and we cannot too often reiterate the precept, that the best rule is "*to obviate occasional symptoms.*" Where the nervous energy is depressed we must stimulate—where the vascular system is greatly excited, and, especially, where any vital organ is threatened, we must deplete locally or generally, according to the state and constitution of the individual. It is worse than madness to lay down a fixed rule, whether of stimulation or depletion, for every case, or for every stage of the same case. The above is the only safe rule—"TREAT SYMPTOMS."

How far the application of caustic to a recent puncture may prevent the acrid matter, in the one case, from producing inflammation of the absorbents—or arrest the progress of the subtle animal poison, in the other case, and prevent it reaching the constitution, we are not prepared to say. We think that the safest plan would be to cleanse the wound immediately, by suction or ablution, and then to destroy its internal parietes by caustic. But after the putrid matter has induced inflammation of the absorbents, or the poison begun to exhibit symptoms of constitutional affection, we cannot conceive the utility of caustic applied to the original wound. Cold saturnine lotions, with opiates at night, and nourishing food, are generally useful in all cases of dissection-wounds where there is much pain—great mental distress—and no great vascular excitement, or appearance of inflammation of an internal organ.

Mr. Higginbottom here introduces several cases illustrative of the good effects of the lunar caustic applied to the wounds made by the bites of animals.

*Bruises.* Mr. H. avers that the caustic is a very valuable application to bruised wounds, especially of the shin—the value of the remedy being greatly enhanced, of course, by its early application. In bruised wounds of the shin, Mr. H. has not had a single instance in which he has not been enabled to effect a cure by the adherent eschar. The difficulty of forming this is always increased by delay, especially on the shin, where there is so much disposition to slough. This slough has always, in Mr. H's practice, been prevented by an early application of the caustic. When the patient applies too late to prevent the sloughing process, we must still treat by caustic, which is to be applied over the bruised and inflamed part. It moderates the inflammation, and when the slough separates, an eschar is to be formed over the exposed sore. Several cases, in illustration, are introduced, for which we must refer to the work.

*Ulcers.* Caustic has long been employed in the cure of ulcers; but, in order to insure its success by eschar, there must be the following conditions—surface not too extensive, nor exposed to friction or motion—nor must there be much discharge. Success will, therefore, be more common in small than in large ulcers, and especially in those irritable and painful little ulcers which are apt to form about the ankle, and occasionally occur near the tendo achillis. In these cases the cold poultice and lotion should precede the caustic for a few days; and after the eschar is formed, the part should be kept exposed to the air and defended from external injury.

The plan of curing ulcers by caustic is exactly that which has been laid down in the treatment by the unadherent eschar. It is necessary, therefore, in all cases, except where the ulcer is very small, to examine the eschar, making a small puncture, or rather a smooth incision in its centre, so as to evacuate the subjacent fluid, if any, taking great care not to break down or bruise the eschar so as to leave its inferior surface at all ragged. This operation is to be repeated daily until the eschar proves to be quite adherent. If the ulcer be rather large, rest should be enjoined, and a saline purgative interposed. Numerous cases are introduced in illustration.

Mr. Higginbottom makes some cursory observations on the treatment, by caustic, of whitlow, inflammation of the finger, fungous ulcer of the navel in infants, inflammation of the knee, tinea capitis, &c. which we must pass over, as we perceive our limits are considerably exceeded. In a short concluding chapter, Mr. H. candidly particularizes those cases in which the caustic would be inapplicable, for he does not, like some modern writers, propose his hobby as a universal carrier. The caustic is inapplicable to recent burns, large ulcers, extensive lacerations, incised wounds, scrofulous sores, erysipelatous inflammation, &c.

We have thus made known the prominent features of Mr. Higginbottom's little work, conscious that in the treatment of the little affections and accidents above alluded to, men are more frequently foiled than in graver surgical diseases. We recollect, some 30 years ago, being asked by an eminent surgeon, who had the charge of examining young gentlemen entering the public service of the state, "if we knew any cure for a sore shin?" This question he asked every one who came before him—as well as most of those with whom he entered into conversation. We thought it, at the time, a mighty silly question; but often since that period, have we recollected the old gentleman's query, and devoutly wished that we, ourselves, had found out the answer to it. Whether the old querist ever obtained the object of his inquiry, we know not, as he has long since been "gathered to his forefathers." Doubtless he would have devoured, with great attention, the pages of Mr. Higginbottom; and it remains for time to prove whether or not our author has at length discovered the long sought "cure for a sore shin." The process by caustic is, as we said before, an imitation of Nature; and we have seen so much utility result from its application in various kinds of irritable sores, that we augur well of Mr. Higginbottom's proposal.

---

